# Investigation on Fatigue Damage Characteristics of Basalt Fiber-Reinforced Asphalt Mixtures with High RAP Content

**DOI:** 10.3390/ma19143057

**Published:** 2026-07-16

**Authors:** Chunfeng Zhu, Zhenyu Wang, Yongyong Yang, Shandong Fang, Yonghong Chen, Bo Xiao, Di Yu

**Affiliations:** 1 College of Civil Engineering, Jilin Jianzhu University, Changchun 130118, China; zhuchunfeng@jlju.edu.cn (C.Z.); tumuyang135@163.com (Y.Y.); 2 Jiujiang Management Center of Jiangxi Communications Investment Group Co., Ltd., Nanchang 330200, China; 3 Jiangxi Provincial Key Laboratory of Traffic Infrastructure Safety, East China Jiaotong University, Nanchang 330013, China; 4 Shangrao Municipal Comprehensive Administrative Law Enforcement Brigade of Transportation, Shangrao 334000, China; 5 Changchun Municipal Engineering Design and Research Institute (Group) Co., Ltd., Changchun 130033, China; bxccsz@163.com

**Keywords:** high-RAP asphalt mixture, basalt fiber, fatigue damage evolution

## Abstract

**Highlights:**

- $R_{CDEC}$ evolution curves of high-RAP mixtures exhibit distinct two-stage damage characteristics.
- Basalt fiber lowers the steady-state energy dissipation rate of high-RAP mixtures.
- BF bridging effects provide a basis for improving brittle defects of high-RAP asphalt matrix.
- BF phase-specific damage control provides technical references for durable pavement design.

**Abstract:**

High-RAP asphalt mixtures are highly susceptible to fatigue brittle fracture. This study investigated the fatigue damage evolution of basalt fiber (BF)-reinforced high-RAP mixtures. Four-point bending fatigue tests were performed to evaluate the stiffness damage factor and the ratio of cumulative dissipated energy change $R_{CEDC}$. The ExpDec2 model and a second-derivative criterion were utilized to quantify steady-state energy dissipation and partition damage stages. Results indicate that BF extended fatigue life, yielding a 55% improvement at 70% RAP content. Model evaluations revealed that in the initial stage, increasing RAP delayed the critical damage inflection point due to accelerated non-steady damage, whereas BF shifted inflection points forward by constraining early damage accumulation. In the stable propagation stage, BF reduced the energy dissipation rates of 50% and 70% RAP mixtures by 44% and 22%, respectively. These quantitative results provide essential performance data regarding the effect of BF on the fatigue life and energy dissipation of high-RAP mixtures, serving as a practical reference for the design and durability optimization of sustainable asphalt pavements.

## 1. Introduction

As a crucial approach to achieving carbon peaking and carbon neutrality goals in pavement engineering [1,2], Central Plant Hot Recycling technology delivers substantial ecological and economic benefits by significantly reducing natural aggregate consumption and minimizing waste asphalt pollution [3,4]. Nevertheless, the practical application of Reclaimed Asphalt Pavement (RAP) is frequently hindered by prominent interfacial heterogeneity, which consequently compromises its field performance [1]. Particularly at high RAP dosages, premature cracking and failure frequently occur. As fatigue cracking constitutes one of the primary distresses in recycled pavements, fatigue testing can directly characterize the stiffness attenuation and damage evolution under cyclic loading. Consequently, enhancing the fatigue resistance of high-RAP asphalt mixtures has become a critical research focus in the field of pavement engineering.

Fibers have long been utilized as a common modifier to improve the performance of recycled asphalt mixtures. Their incorporation not only enhances the high- and low-temperature properties and moisture stability of the mixtures, but also effectively extends their fatigue life and load-bearing capacity [5,6]. Among these, basalt fiber (BF) is widely favored by researchers owing to its exceptional physical properties and environmental sustainability. It exhibits distinct advantages in retarding crack propagation within the recycled asphalt matrix and enhancing overall performance [7,8,9,10,11]. Oral [12] compared the mechanical properties of basalt and ceramic fibers, determining the optimal content for both. Chen [13] evaluated the optimal dosage of three types of fibers in high-modulus asphalt mixtures through a series of laboratory tests. Wang [14] demonstrated that basalt fiber significantly extends the fatigue life of mixtures, indicating that fibers mitigate stress concentration in critical zones and thereby reduce fatigue damage. Sun [15] suggested that fibers could effectively enhance the structural strength of aged asphalt, although its wear resistance tends to decrease over time.

To accurately characterize complex fatigue behavior, researchers have introduced various mathematical models to conduct quantitative investigations. Yin [16] established a neural network model that demonstrated high fitting accuracy, indicating that fatigue and fatigue limit equations incorporating a temperature factor can better describe the coupling relationship between RAP and fatigue performance. Zhang [17] applied viscoelastic continuum damage (VECD) mechanics to study the fatigue behavior of asphalt mixtures, developing a comprehensive set of fatigue life prediction equations. Lu [18] evaluated the fatigue performance of three types of fibers in RAP mixtures and derived calculation equations for the cumulative stiffness modulus degradation ratio (CSMDR) of asphalt mixtures under different strain levels. Song [19] proposed that the minimum ratio of cumulative dissipated energy change ($R_{CDEC-\min}$) could serve as a universal indicator for fatigue performance. Shu et al. [20] conducted four-point bending fatigue tests and demonstrated the validity of the energy ratio concept in the field of fatigue cracking, noting that fatigue cracking resistance can also be quantified via fracture mechanics principles. Teixeira et al. [21] further investigated the effects of aggregate gradation and binder type on the fatigue life of hot mix asphalt (HMA) through cyclic semi-circular bending (SCB) tests, suggesting that the SCB test can serve as a rapid and effective method to evaluate HMA fatigue characteristics. Liu [22] identified the damage law of high-RAP asphalt mixtures based on a non-linear creep-fatigue damage model, thereby optimizing the fatigue life prediction model and analyzing the parameters influencing the actual fatigue state of pavements.

In summary, despite extensive research using non-linear damage equations and energy models, current studies rely excessively on macroscopic phenomenological trends or global empirical formulas, thereby obscuring the highly non-linear and multi-stage characteristics of fatigue evolution within severely embrittled matrices. Within the existing framework, research utilizing the ratio of cumulative dissipated energy change ($R_{CDEC}$) to analyze the fatigue properties of fiber-reinforced recycled asphalt mixtures is limited, particularly regarding the application of non-linear models for the quantitative segmentation of the fatigue process. Given that traditional organic fibers function primarily as oil-absorbent stabilizers, their reinforcement and crack-bridging efficacy within severely embrittled high-reclaimed-asphalt-pavement (high-RAP) matrices is restricted. Conversely, basalt fiber (BF), as a high-modulus inorganic reinforcing material, exhibits distinct mechanical advantages. This study evaluates BF-reinforced recycled asphalt mixtures to determine the specific fatigue stages and exact percentages by which the fiber suppresses damage accumulation in high-RAP mixtures.

Therefore, this study compares recycled asphalt mixtures incorporated with basalt fibers, introducing a refined quantitative method to analyze the multi-stage fatigue evolution laws of fiber-reinforced recycled asphalt mixtures. The specific research and procedures are as follows:

Recycled asphalt mixtures containing basalt fibers were prepared, with the fiber-free mixtures serving as the control group, to perform four-point bending fatigue tests, systematically summarizing the stiffness damage factor ($D_{S}$) and the ratio of cumulative dissipated energy change ($R_{CDEC}$) under cyclic loading. By combining the ExpDec2 non-linear model with a mathematical second-derivative criterion, the critical damage inflection points were determined to achieve a precise digital measurement of the efficiency of BF in suppressing the steady-state energy dissipation rate. This work aims to construct an energy-based multi-stage fatigue evaluation framework, thereby providing crucial theoretical support for the durability design and performance optimization of sustainable high-RAP pavements.

## 2. Materials and Methods

### 2.1. Materials

#### 2.1.1. Basalt Fiber

**Figure 1:**
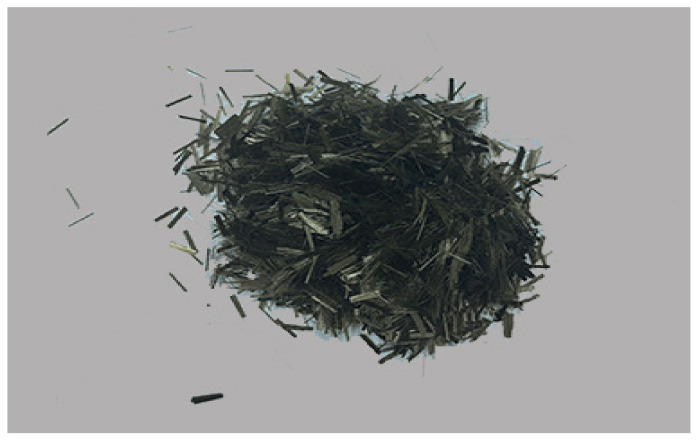
Fiber sample.

The 6 mm chopped basalt fiber utilized in this study, as shown in Figure 1, was supplied by Jilin Tongxin Basalt Technology Co., Ltd. (Jilin, China). Based on a previous study [23], the optimal dosage was selected as 0.3%, and its primary technical parameters are summarized in Table 1.

#### 2.1.2. Rheological Optimization of Rejuvenator

In this study, Evoflex 8182, a rejuvenator manufactured by Ingevity Corporation (North Charleston, SC, USA), was utilized. Its basic technical specifications are shown in Table 2. Based on Bending Beam Rheometer (BBR) and Dynamic Shear Rheometer (DSR) tests, the optimal dosage of the rejuvenator was determined to be 5%.

#### 2.1.3. New Aggregates and Virgin Asphalt

In this study, obtained from a local construction site in Nanchang (Jiangxi, China), was utilized as both the coarse and fine aggregates due to its hard texture and clean surface. Limestone mineral powder was selected as the filler, which was dry, uniform, and free of agglomeration. Aggregate performance testing was conducted in accordance with the Test Methods of Aggregate for Highway Engineering [24], with all results satisfying the specified criteria. The virgin binder used was an SBS-modified asphalt, whose conventional physical properties complied with the relevant requirements in the Technical Specifications for Construction of Highway Asphalt Pavements [25]. The specific technical indicators of the SBS-modified asphalt are detailed in Table 3.

#### 2.1.4. Reclaimed Asphalt Pavement (RAP)

The RAP utilized in this study was sourced from an in-service highway in Jiangxi Province, recovered via mechanical milling, and subsequently pretreated using a crusher and a vibrating screen. Based on its particle size distribution, the RAP was fractionated into three distinct grades: Grade A (0–8 mm), Grade B (8–12 mm), and Grade C (12–22 mm). In accordance with the Test Methods of Aggregate for Highway Engineering [24], the RAP was subjected to extraction to separate the reclaimed aggregates and aged asphalt. Their relevant physical and technical indicators are summarized in Table 4.

#### 2.1.5. Mixture Design

**Figure 2:**
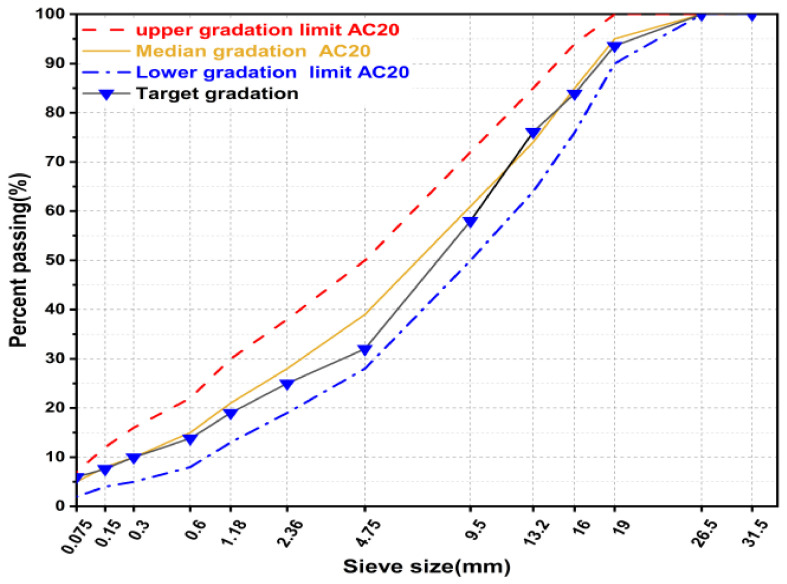
Aggregate gradation curves.

The aggregate gradation curve in this study was determined according to the Chinese specification JTG F40-2004 [25]. To eliminate compounding effects induced by fiber incorporation, an identical gradation curve was adopted, as shown in Figure 2. The mix design of the basalt-fiber-reinforced recycled asphalt mixture was conducted using the Marshall design method, and the optimum asphalt content (OAC) was determined accordingly. Additionally, gray relational analysis (GRA) was employed to optimize the three fractions of RAP. The optimization outcomes and the specific OAC for each mixture group are summarized in Table 5 and Table 6, respectively.

### 2.2. Experiments and Methods

#### 2.2.1. Four-Point Bending Fatigue Test

**Figure 3:**
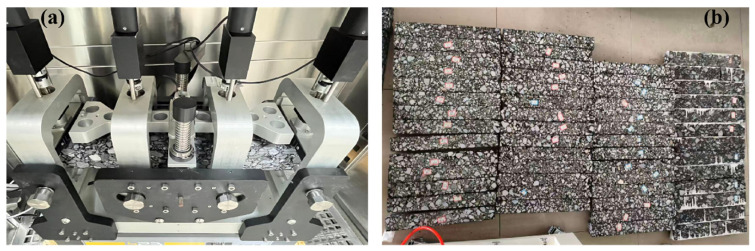
Fatigue test apparatus and specimen: (**a**) test setup, (**b**) fatigue specimen.

In this study, specimens were prepared in accordance with the specifications in [26], with the compaction effort set to 24 round-trip passes. The compacted slabs were subsequently saw-cut into five standard four-point bending fatigue specimens with dimensions of 380 mm × 63.5 mm × 50 mm. Figure 3a,b illustrate the fatigue test apparatus and the prepared specimen, respectively. For each mixture group, three replicate specimens were selected to conduct the parallel tests.

The fatigue tests were performed using a standard four-point bending test system (IPC Global, Melbourne, Australia) in a strain-controlled mode at a strain level of 600 με and a temperature of 20 °C, utilizing a 10 Hz haversine loading waveform. The stiffness modulus at the 50th load cycle was defined as the initial value, and the test was terminated when the stiffness modulus degraded to 50% of its initial counterpart.

#### 2.2.2. Stiffness Degradation Evaluation Based on Damage Factor $D_{S}$

Considering the inherent scatter of fatigue test data, representative specimens with fatigue lives close to the group mean were selected from each parallel group for analysis. Based on continuum damage theory [27], the flexural stiffness modulus damage factor $D_{S}$ was introduced to evaluate the fatigue attenuation characteristics of various recycled asphalt mixtures. The calculation is expressed in Equation (1):

(1) $$D_{S}=1-\frac{S_{i}}{S_{0}}$$

where $S_{0}$ is the initial reference stiffness modulus (fixed at the 50th loading cycle to serve as the control baseline value for the undamaged state of the material, MPa); and $S_{i}$ is the dynamic real-time stiffness modulus recorded at the current i-th loading cycle (dynamically varying cycle by cycle with the evolution of fatigue damage, MPa).

#### 2.2.3. Fatigue $R_{CDEC}$ Evolution Equation and Regression Modeling

To eliminate initial energy variations among specimens and precisely capture the dynamic characteristics of the damage rate, the ratio of cumulative dissipated energy change $R_{CDEC}$ was introduced [28] as an evaluation indicator for fatigue cracking susceptibility. The dissipated energy at the N load cycle and the calculation of $R_{CDEC}$ are formulated in Equations (2) and (3), respectively:

(2) $$C_{DE\_N}=\sum_{K=1}^{N}D_{E,K}$$

(3) $$R_{CDEC}=\frac{C_{DE\_N+1}-C_{DE\_N}}{C_{DE\_N}}$$

**Figure 4:**
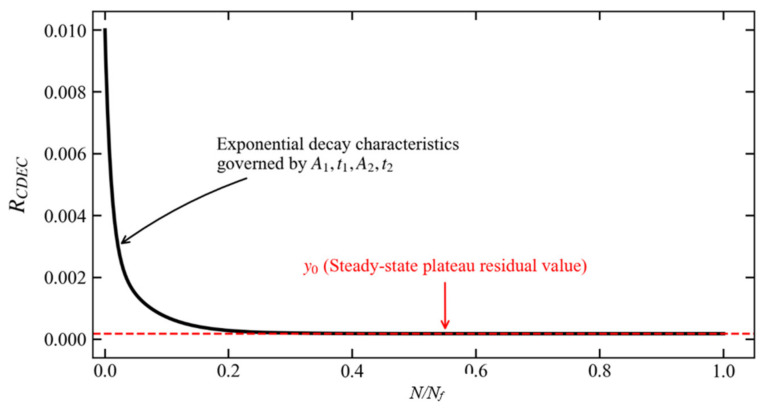
Schematic representation of the $R_{CEDC}$ double-exponential model.

To mathematically characterize the continuous attenuation and regression behavior of $R_{CDEC}$ throughout the fatigue process, a double-exponential decay model (ExpDec2), inspired by the Prony series representation in viscoelastic mechanics [29], was introduced as expressed in Equation (4). The regression modeling was performed utilizing OriginPro 2021 (OriginLab, Northampton, MA, USA).

(4) $$R_{CDEC}=y_{0}+A_{1}e^{-\frac{x}{t_{1}}}+A_{2}e^{-\frac{x}{t_{2}}}$$

where $y_{0}$ represents the steady-state plateau residual value; $A_{1}$ and $A_{2}$ denote the fitting parameters associated with the damage amplitude; and $t_{1}$ and $t_{2}$ are the characteristic constants corresponding to the mechanical damage stages. To visually clarify the geometric and physical significance of these regression terms throughout the fatigue process, a typical regression profile of the $R_{CEDC}$ evolution curve is illustrated in Figure 4.

#### 2.2.4. Damage Stage Partitioning Based on the Second Derivative of the Evolution Equation

To achieve a refined quantification of the phased characteristics of fatigue damage, this study defines a “Damage Inflection Point” based on the geometric features of the dissipated energy evolution curve. The second derivative of the evolution equation is introduced as the mathematical criterion for stage partitioning, with the threshold for identifying the DIP established where the second derivative falls below 10^−2^. This inflection point is defined as the critical transition point where the curve shifts from an initial rapid non-linear decay to a stable state with a constant slope, serving as the physical and mathematical boundary that separates the early rapid damage (Stage I) from the mid-term stable energy dissipation (Stage II) of the specimens. Additionally, the mean values of the Stage II span for different types of recycled hot-mix asphalt are calculated to analyze the reinforcing mechanisms of fibers during this low-rate damage evolution phase. Based on these theoretical foundations and evaluation indicators, a comprehensive fatigue evolution analysis framework centered on the double-exponential decay model is established. The subsequent sections will discuss the evolution laws of fatigue damage in detail, integrating the specific experimental results.

## 3. Results and Discussion

### 3.1. Variation of Flexural Stiffness Modulus with Loading Cycles

**Figure 5:**
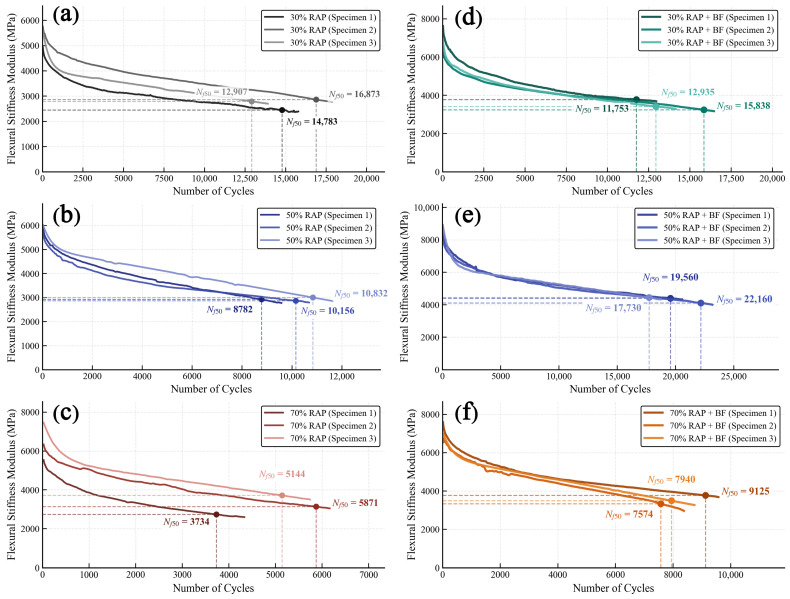
Flexural stiffness modulus decay curves of different asphalt mixtures: (**a**) 30% RAP; (**b**) 50% RAP; (**c**) 70% RAP; (**d**) 30% RAP + BF; (**e**) 50% RAP + BF; (**f**) 70% RAP + BF.

Figure 5 illustrates the evolution curves of the flexural stiffness modulus for recycled asphalt mixtures with various RAP contents. Specifically, Figure 5a–c present the control mixtures with 30%, 50%, and 70% RAP content without fibers, respectively, while Figure 5d–f depict the corresponding fatigue test results for the mixtures reinforced with BF.

Overall, from the perspective of the variation in the flexural stiffness modulus curves, the initial stiffness modulus of the mixture increases with the increment of RAP content, which is consistent with the findings reported by Mateos et al. [30]. However, its fatigue life exhibits a sharp reduction. This degradation is primarily attributed to the substantial amount of severely aged asphalt binder in the high-RAP mixtures, which imparts a distinct brittle and stiff nature to the overall material. Under cyclic loading, microcracks within the matrix are highly prone to unstable propagation and coalescence, resulting in premature fatigue failure. Conversely, the incorporation of fibers significantly extends the fatigue life, demonstrating that BF constructs an effective three-dimensional spatial reinforcing network inside the mixture. Comparative analysis reveals that the 50% RAP + BF group exhibits the optimal performance; it successfully retains a relatively high initial stiffness while effectively mitigating the degradation rate of the flexural stiffness modulus. This indicates that an appropriate RAP dosage can form a favorable synergistic effect with BF. Notably, although the introduction of BF at the 70% RAP content improves the fatigue life compared to the unreinforced counterpart, its absolute fatigue life remains significantly lower than that of the unreinforced 50% RAP control group. This phenomenon implies that when the RAP content is excessively high, the brittle failure characteristics governed by the aged binder become overwhelmingly dominant, which severely restricts the spatial reinforcing efficiency of BF. Consequently, from the perspective of fatigue durability, the compromised fatigue life of recycled hot-mix asphalt under high RAP contents is difficult to resolve solely through the external blending of BF.

### 3.2. Analysis of Flexural Stiffness Modulus Damage Factor

To further elucidate the underlying damage accumulation mechanisms governing the aforementioned macroscopic fatigue life and stiffness variations, the flexural stiffness modulus damage factor ($D_{S}$) was introduced to analyze the dynamic fatigue damage evolution processes of various mixtures.

**Figure 6:**
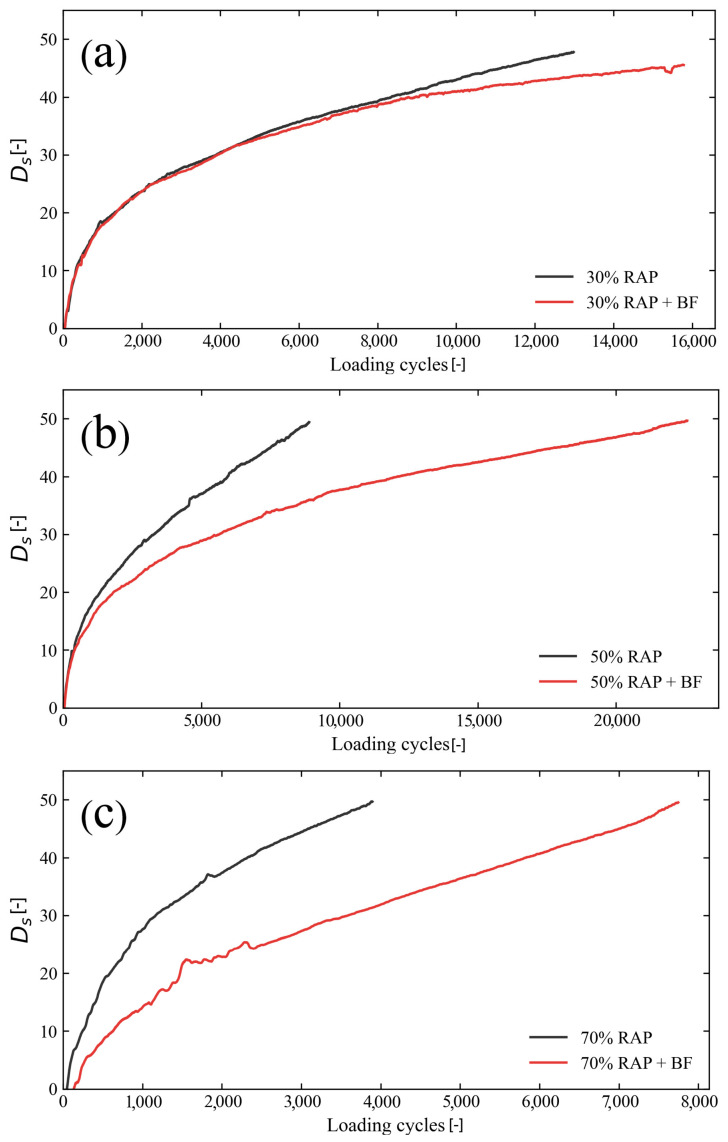
Stiffness damage factor growth curves: (**a**) 30% RAP; (**b**) 50% RAP; (**c**) 70% RAP.

Figure 6 illustrates the effect of BF on the flexural stiffness modulus damage under different RAP contents. It can be clearly observed that the fatigue damage process of the recycled hot-mix asphalt exhibits non-linear evolution with distinct two-stage characteristics. In Stage I, the $D_{S}$ value increases rapidly, indicating an accelerated accumulation of specimen damage. In Stage II, the $D_{S}$ value enters a stable propagation phase, and the damage growth tends to level off.

Specifically, at the 30% RAP content (Figure 6a), the damage accumulation curves of the unreinforced recycled group and the BF-reinforced group exhibit a high degree of overlap with virtually identical evolution slopes, suggesting that the inhibitory effect of BF on damage propagation is insignificant at this low dosage. At the 50% RAP content (Figure 6b), the damage factor of the BF-containing specimens remains substantially lower than that of the fiber-free group throughout the fatigue evolution cycle, and the slope during the mid-term stable damage phase is drastically reduced. This demonstrates that the toughening effect of BF significantly extends the service life of the material. However, at the high RAP content (70% RAP, Figure 6c), although the incorporation of BF suppresses the catastrophic, precipitous failure observed in the unreinforced control group, its improvement remains limited by the severe aging and embrittlement of the asphalt binder [31]. Consequently, the growth rate of $D_{S}$ in Stage II for the 70% RAP + BF group is much greater than that for the 50% RAP + BF group as Figure 6b, indicating that the modification efficiency of BF is relatively compromised at excessive RAP dosages.

In summary, the incorporation of BF exerts a distinctly positive effect on mitigating the fatigue damage evolution process of recycled hot-mix asphalt across various RAP contents. This mitigation is specifically manifested in suppressing early-stage damage release, extending the mid-term stable propagation stage, and delaying the ultimate propagation of fatigue failure.

### 3.3. Analysis of R_CDEC_ Evolution Laws

**Figure 7:**
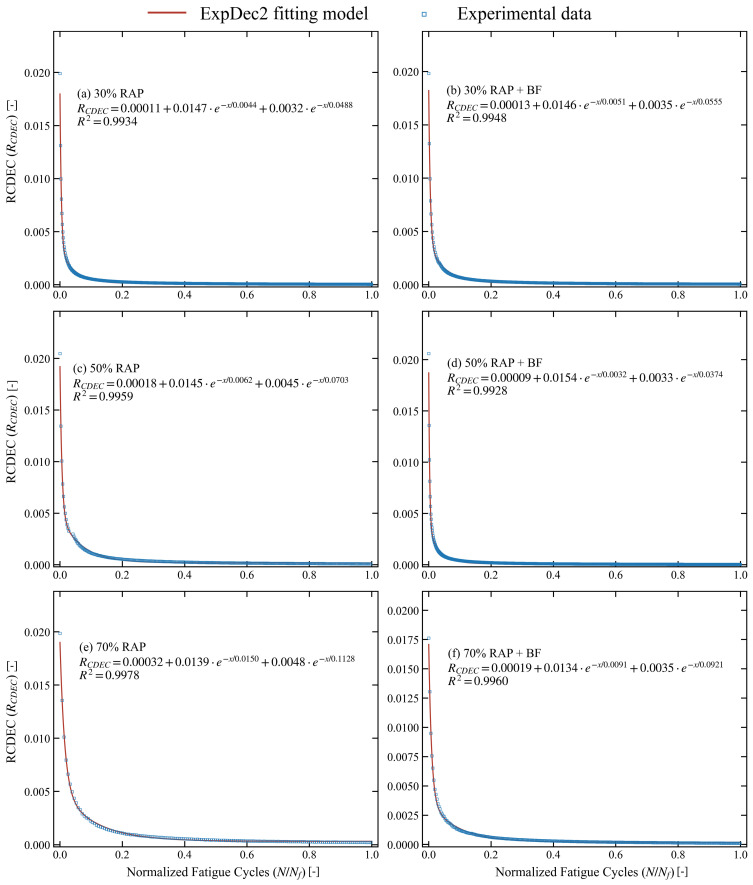
ExpDec2 model fitting results of RCEDC evolution curves for different asphalt mixtures: (**a**) 30% RAP; (**b**) 30% RAP + BF; (**c**) 50% RAP; (**d**) 50% RAP + BF; (**e**) 70% RAP; (**f**) 70% RAP + BF.

To precisely quantify the non-linear fatigue damage process of the mixtures with various RAP contents, the ratio of cumulative dissipated energy change $R_{CDEC}$ was introduced, and the ExpDec2 double-exponential decay model was employed to conduct non-linear regression fitting on its evolution against the normalized fatigue life ($N/N_{f}$). The fitting curves for each test group are illustrated in Figure 7, and the corresponding governing equations and parameters are tabulated in Table 7.

Horizontal comparison of the fitting equations for the unreinforced control groups (a), (c), and (e) in Figure 7 reveals that with the increase of RAP content, the steady-state residual constant exhibits a significant increasing trend. Therefore, it can be concluded that after the high-content RAP mixture enters the second stage (stable damage period), the plateau position of its curve is obviously “raised” as a whole. This objectively reflects that after the brittleness and stiffness of the material increase, the “basic energy dissipation rate” required to maintain its own stability under the same cyclic load increases significantly.

Conversely, comparing the corresponding groups before and after fiber incorporation reveals significant changes in the fitting equations. Taking the 50% content as an example, its value decreases sharply after the incorporation of BF; at the 70% content, the addition of BF similarly reduces the value from 0.00032 to 0.00019. This indicates that the fiber incorporation stabilizes it at a lower baseline, demonstrating that the reinforcing effect of fibers reduces the energy dissipation of the mixture during the propagation period.

In conclusion, the energy dissipation characteristics of recycled hot-mix asphalt during the fatigue steady-state period are dually governed by the RAP aging degree and the reinforcing effect of BF. Specifically, a high RAP content significantly aggravates the internal energy dissipation burden of the brittle matrix, whereas the three-dimensional network of BF counteracts this negative effect, thereby effectively extending the steady-state life. Furthermore, the constant term ($y_{0}$) monotonically increases with higher RAP content, reflecting that binder aging elevates the baseline damage, whereas the addition of basalt fibers significantly lowers $y_{0}$, proving that the 3D fiber network effectively reduces steady-state damage accumulation.

### 3.4. Determination of Damage Inflection Point Based on $R_{CDEC}$ Evolution Equation

**Figure 8:**
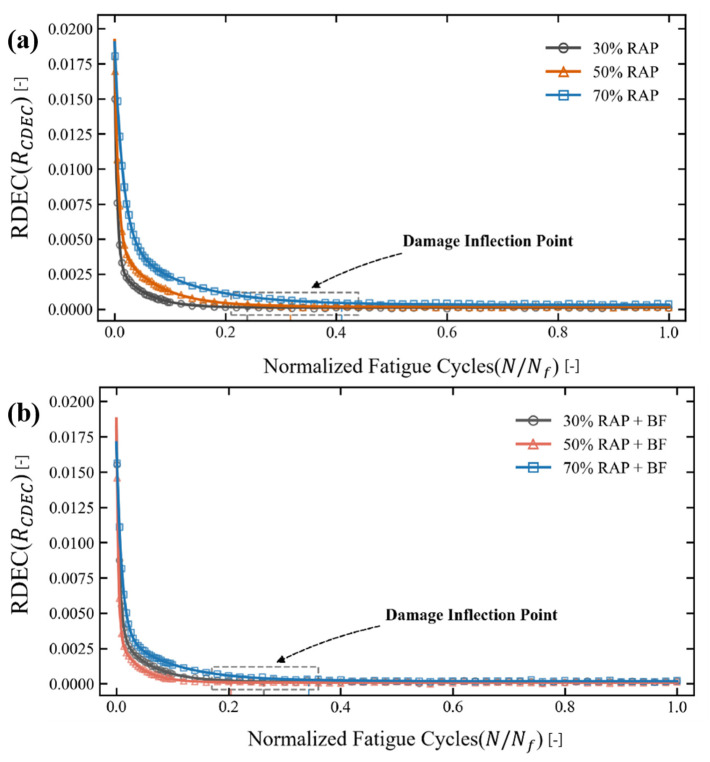
Determination of RCEDC damage inflection points for different asphalt mixtures: (**a**) control group without fiber; (**b**) group modified with BF. (the DIP is mathematically determined by the second derivative threshold of 10^−2^ as detailed in Section 2.2.4).

The aforementioned model fitting results validate the soundness of the double-exponential decay law as a whole and reveal the macroscopic trend of energy dissipation evolution. To determine the physical boundary between Stage I and Stage II, the fatigue damage evolution inflection points of the specimens were determined, as shown in Figure 8.

Figure 8a illustrates the fatigue damage evolution laws of the unreinforced control groups with different RAP contents. It can be clearly observed from the figure that with the increase of RAP content, the $R_{CDEC}$ curves shift upward as a whole, indicating that the early damage evolution rate of the recycled hot-mix asphalt increases significantly, and the fatigue resistance of the mixture decreases. Specifically, the damage inflection point of the 30% RAP mixture occurs at $N/N_{f}=0.27$, while those of the 50% RAP and 70% RAP mixtures are located at 0.32 and 0.36, respectively. This trend demonstrates that a higher RAP content leads to greater early damage accumulation, thereby delaying the entry into the low-damage steady propagation stage. The primary reason is that as the RAP content increases, the proportion of severely aged asphalt binder in the mixture rises substantially, which compromises the blending and bonding efficiency at the virgin-aged asphalt interface. Consequently, under early cyclic loading, larger new micro-damages continuously initiate within the matrix.

Figure 8b further illustrates the evolution characteristics of the recycled hot-mix asphalt with BF under different RAP contents. Compared with the unreinforced control groups, the damage inflection points of the fiber-reinforced mixtures all shift forward, indicating that BF can effectively suppress the rapid evolution of early damage in the recycled hot-mix asphalt, thereby enhancing its fatigue resistance. Regarding the mixtures with different RAP contents, the damage inflection point of 30% RAP + BF shifts slightly forward to $N/N_{f}=0.21$; that of 50% RAP + BF shifts forward to $N/N_{f}=0.22$, yielding the optimal improvement effect; and that of 70% RAP + BF shifts forward to $N/N_{f}=0.33$. This is attributed to the three-dimensional spatial network structure formed by BF, which effectively constrains the initiation and propagation of microcracks during the early fatigue damage process of the specimens.

### 3.5. Fatigue Damage Trend Analysis Based on $R_{CDEC}$ Stage Characteristics

**Figure 9:**
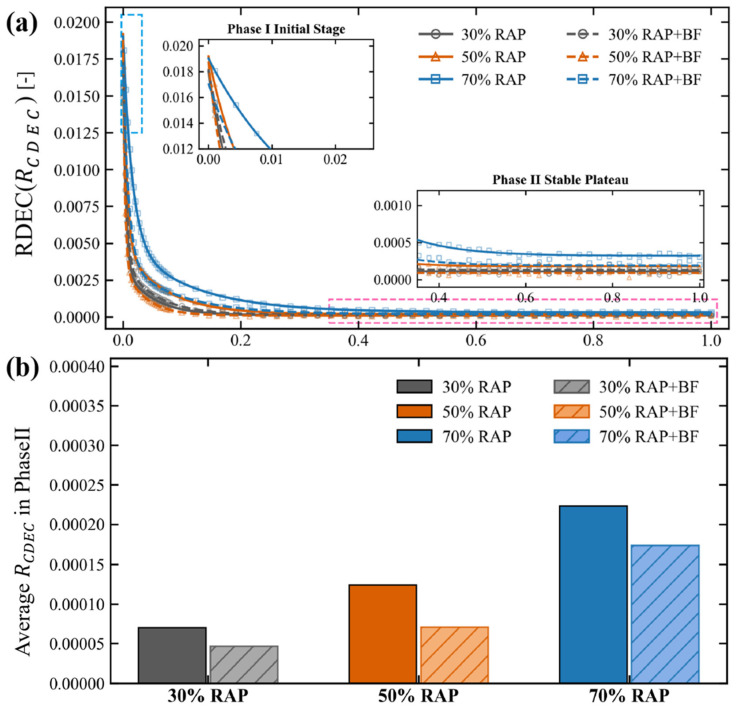
$R_{CEDC}$ fatigue evolution characteristics of different mixtures: (**a**) evolution curves over the entire life cycle, (**b**) mean values of steady-state energy dissipation in Phase II.

Based on the experimental data, this section performs a quantitative analysis on the unsteady and steady-state characteristics of the fatigue damage stages for different recycled hot-mix asphalts. Figure 9 illustrates the effects of various RAP contents and BF on the evolution curves.

As observed from Figure 9a (Phase I Initial Stage), during Phase I (initial loading stage), the curves shift upward significantly and extend to the right with the increment of RAP content, indicating that the strong heterogeneity of the aged asphalt accelerates the early unsteady damage accumulation rate of the material. Conversely, after the incorporation of BF, the curves of all groups markedly converge toward the lower left, demonstrating that the introduction of fibers can effectively constrain the early stiffness damage and suppress the initial unsteady energy release. Upon entering the Phase II Stable Plateau, the unreinforced groups exhibit a stepwise decrease with the increase of RAP content, implying that a higher RAP content leads to a higher damage rate. After the incorporation of BF, the curves of all groups shift downward, verifying the fatigue resistance enhancement of the fibers under long-term loading.

Figure 9b further presents the mean value variations during Phase II. As can be observed, for the unreinforced control groups, the mean steady-state energy dissipation increases in a stepwise manner as the RAP content rises from 30% to 70%. This is primarily because the significantly increased proportion of aged asphalt at high RAP contents enhances the brittleness and stiffness of the mixture, thereby accelerating the unit damage rate during the stable evolution stage. Upon the incorporation of BF, the mean steady-state values of all groups exhibit a systematic downward shift, in which the mean steady-state energy dissipation of the 50% RAP and 70% RAP groups is significantly reduced by 44% and 22%, respectively, with the 50% RAP group exhibiting the most pronounced reduction. These findings confirm that the three-dimensional spatial network structure formed by BF exerts excellent crack-bridging, crack-restraining, damage-control, and energy-mitigation effects under long-term cyclic loading, effectively reducing the energy dissipation rate during the stable service period, thereby delaying the overall fatigue failure process of the mixture.

## 4. Conclusions

Regarding the fatigue performance of recycled asphalt mixtures, this paper carried out four-point bending fatigue tests and systematically analyzed the effects of different RAP contents and basalt fiber (BF) on the non-linear evolution laws of macro-life, stiffness damage factor, and ratio of cumulative dissipated energy change ($R_{CDEC}$) of the mixtures. The following conclusions are drawn:

(1) Basalt fiber can significantly improve the fatigue cracking resistance of high-content recycled matrices. Relying on its high elastic modulus, it exerts its excellent physical bridging mechanism during loading. With the increase of RAP content, the aged asphalt leads to an increase in the overall brittleness of the mixture, and the initial flexural tensile stiffness modulus significantly rises, but the absolute fatigue life undergoes sharp attenuation. After incorporating basalt fiber, the improvement is most obvious at 50% content. At 70% content, fiber reinforcement increases its fatigue life with an increase rate of 67.5%. At 30% content, no significant life extension is observed.

(2) The damage accumulation of the mixture presents obvious non-linear two-stage characteristics. With the increase of RAP content, the $R_{CDEC}$ curves of mixtures without fiber shift upward as a whole, the damage inflection point is delayed, and the damage factor changes extremely fast in the first stage, while the damage growth rate in the second stage is high and the stable period is short. After incorporating basalt fiber, the damage inflection points of all RAP groups show a forward shift, and the damage accumulation rate in the second stage is substantially reduced. It is confirmed that the network structure formed by fibers inside the mixture effectively delays the propagation of fatigue failure.

(3) Based on the stiffness modulus damage factor and $R_{CDEC}$ analysis, the higher the RAP content, the more severe the damage evolution of the mixture in the initial and stable stages. The addition of basalt fiber not only effectively constrains the early damage accumulation, but also significantly reduces the energy dissipation rate in the stable stage, and the damage control effect is most significant at 50% RAP.

In summary, basalt fiber can effectively improve the fatigue cracking resistance of recycled asphalt mixtures, which can not only delay the stiffness damage accumulation speed in the early stage of loading, but can also reduce the energy dissipation rate in the mid-to-late stable stage, providing an effective reference for fiber-reinforced recycled asphalt mixtures.

## Figures and Tables

**Table 1:** Technical parameters of basalt fiber [23].

Test Items	Unit	Test Results	Technical Requirements
Appearance	%	98.6	≥90
Fiber length	mm	6.00	6 ± 10%
Linear density	tex	225.1	225.8 ± 8%
Fiber diameter	mm	17.01	17 ± 10%
Combustible	——	Non-flammable in open flame	Non-flammable in open flame
Density	g/cm^3^	2.66	2.60–2.80
Modulus of elasticity	Mpa	81.5 × 10^3^	≥7.5 × 10^3^
Elongation at break	%	2.4	2.4–3.1
Fracture strength	Mpa	1675	1200–2200
Moisture content	%	0.036	≤0.2

**Table 2:** Evoflex8182 basic technical specifications [23].

Test Items	Unit	Test Value	Standard
Viscosity at 60 °C	cst	9532	T0619 [24]
Flash point	°C	240.4	T0633 [24]
Saturates content	%	21.5	T0618 [24]
Aromatic content	%	41.5	T0618
Viscosity ratio before thin film oven test	——	1.3	T0619
Mass change before and after thin film oven test	%	0.8	T0609 [24]

**Table 3:** Basic Technical Properties of SBS-Modified Asphalt [23].

Test Items	Unit	Standard	Test Value	Specification Value
Penetration	0.1 mm (@25 °C, 100 g, 5 s)	T0604 [24]	58.5	30–60
Ductility	cm (@5 °C, 5 cm/min)	T0605 [24]	31.2	≥20
Softening point	°C	T0606 [24]	78.8	≥60
Viscosity at 135 °C	Pa·s	T0625 [24]	2.93	≤3

**Table 4:** Basic Technical Properties of RAP.

Test Indicators/Mean Values	Recycled Old Asphalt			Regulatory Requirements	Test Standard
	Grade A	Grade B	Grade C		
25 °C penetration (0.1 mm)	29.8	33.1	31.6	≥20	T0604
Softening Point (°C)	72.5	70.1	71.6	actual measurement	T0606
Ductility (15 °C) (cm)	3.8	5.6	4.8	actual measurement	T0605

**Table 5:** Optimization Results of Grey Relational Grade.

RAP Blending Ratio (%)	Type of RAP		
	0–8 mm/%	8–12 mm/%	12–22 mm/%
30	6	11	13
50	10	19	21
70	14	26	30

**Table 6:** Optimization results of total and virgin asphalt content for mixtures.

Asphalt Content	Mixture Type							
	0%	0% (BF)	30%	30% (BF)	50%	50% (BF)	70%	70% (BF)
Optimal total asphalt content (%)	4.0	4.29	4.05	4.38	4.40	4.72	4.61	4.89
Optimal virgin asphalt content (%)	—	—	2.78	3.11	2.28	2.6	1.65	1.93

**Table 7:** ExpDec2 model fitting parameters for $R_{CDEC}$ evolution curves of different recycled asphalt mixtures.

Mixture Type	Fitting Equation	Goodness of Fit/R^2^
(a) 30%RAP	$R_{CDEC}=0.00011+0.0147\cdot e^{-\frac{N/N_{f}}{0.0044}}+0.0032\cdot e^{-\frac{N/N_{f}}{0.0488}}$	0.99
(b) 30%RAP + BF	$R_{CDEC}=0.00013+0.0146\cdot e^{-\frac{N/N_{f}}{0.0051}}+0.0035\cdot e^{-\frac{N/N_{f}}{0.0555}}$	0.99
(c) 50%RAP	$R_{CDEC}=0.00018+0.0145\cdot e^{-\frac{N/N_{f}}{0.0062}}+0.0045\cdot e^{-\frac{N/N_{f}}{0.0703}}$	0.99
(d) 50%RAP + BF	$R_{CDEC}=0.00009+0.0154\cdot e^{-\frac{N/N_{f}}{0.0032}}+0.0033\cdot e^{-\frac{N/N_{f}}{0.0374}}$	0.99
(e) 70%RAP	$R_{CDEC}=0.00032+0.0139\cdot e^{-\frac{N/N_{f}}{0.0035}}+0.0033\cdot e^{-\frac{N/N_{f}}{0.1128}}$	0.99
(f) 70%RAP + BF	$R_{CDEC}=0.00019+0.0134\cdot e^{-\frac{N/N_{f}}{0.0091}}+0.0035\cdot e^{-\frac{N/N_{f}}{0.0821}}$	0.99

## Data Availability

The original contributions presented in this study are included in the article. Further inquiries can be directed to the corresponding author.
